# Drug-Loaded Bioscaffolds for Osteochondral Regeneration

**DOI:** 10.3390/pharmaceutics16081095

**Published:** 2024-08-21

**Authors:** Yifan Tong, Jiaqi Yuan, Zhenguang Li, Cuijun Deng, Yu Cheng

**Affiliations:** Shanghai Key Laboratory of Anesthesiology and Brain Functional Modulation, Clinical Research Center for Anesthesiology and Perioperative Medicine, Translational Research Institute of Brain and Brain-like Intelligence, Shanghai Fourth People’s Hospital, School of Medicine, Tongji University, Shanghai 200434, China; 2211437@tongji.edu.cn (Y.T.); 2211438@tongji.edu.cn (J.Y.); lizhenguang@tongji.edu.cn (Z.L.)

**Keywords:** drug-loaded bioscaffold, 3D printing, osteoarthritis, cartilage repair, osteochondral regeneration

## Abstract

Osteochondral defect is a complex tissue loss disease caused by arthritis, high-energy trauma, and many other reasons. Due to the unique structural characteristics of osteochondral tissue, the repair process is sophisticated and involves the regeneration of both hyaline cartilage and subchondral bone. However, the current clinical treatments often fall short of achieving the desired outcomes. Tissue engineering bioscaffolds, especially those created via three-dimensional (3D) printing, offer promising solutions for osteochondral defects due to their precisely controllable 3D structures. The microstructure of 3D-printed bioscaffolds provides an excellent physical environment for cell adhesion and proliferation, as well as nutrient transport. Traditional 3D-printed bioscaffolds offer mere physical stimulation, while drug-loaded 3D bioscaffolds accelerate the tissue repair process by synergistically combining drug therapy with physical stimulation. In this review, the physiological characteristics of osteochondral tissue and current treatments of osteochondral defect were reviewed. Subsequently, the latest progress in drug-loaded bioscaffolds was discussed and highlighted in terms of classification, characteristics, and applications. The perspectives of scaffold design, drug control release, and biosafety were also discussed. We hope this article will serve as a valuable reference for the design and development of osteochondral regenerative bioscaffolds and pave the way for the use of drug-loaded bioscaffolds in clinical therapy.

## 1. Introduction

Osteochondral defect is a condition characterized by the presence of lesions in both the articular hyaline cartilage and the subchondral bone, commonly caused by trauma or osteoarthritis [1,2]. When osteochondral defects occur, patients typically suffer from joint pain, stiffness, and deformity. These symptoms can lead to severe complications, including a reduced range of motion and joint dysfunction. Ultimately, these conditions can significantly diminish the patient’s quality of life [3]. It has been reported that approximately 250 million people worldwide over the age of 50 suffer from osteoarthritis, with the average annual cost per person estimated to be USD 700 to USD 1560 (in 2019 USD) [4,5]. Amid this considerable economic burden, the majority of osteoarthritis patients with osteochondral defects fail to receive adequate treatment. Given that osteoarthritis is a complex chronic condition, it leads to the gradual and often undetectable progression of osteochondral defects. The typical progression of osteochondral defects involves the following stages: initially, the inflammatory microenvironment within the articular cavity triggers the degradation of the extracellular matrix in articular cartilage. This degradation results in the destruction of the collagen network surrounding chondrocytes, disrupting chondrocyte metabolism, causing abnormal differentiation, and potentially leading to apoptosis. Ultimately, these processes culminate in significant cartilage damage. Due to the strong integration of natural structures, defects in the cartilage often extend into the subchondral bone, evolving into more complex osteochondral defects [6]. It is precisely because osteochondral defects involve cartilage and subchondral bone, which exhibit marked differences in composition, structure, and physiological functions, that the treatment of osteochondral defects represents a significant clinical challenge.

Currently, clinical treatment strategies for osteochondral defects include microfracture, autologous/allogeneic tissue transplantation, and autologous chondrocyte implantation (ACI). Although these treatments are widely utilized in clinical practice, each of them has its own drawbacks. For instance, microfracture often results in the formation of fibrocartilage, which possesses inferior mechanical properties [7,8]. The application of autologous or allogeneic osteochondral transplantation is constrained by the restricted availability of donors’ sources and the potential risk of postoperative infections [9,10]. ACI requires multiple surgeries, imposing a significant burden to patients [11]. Therefore, the current clinical treatments frequently encounter issues such as limited applicability or unsatisfactory long-term therapeutic outcomes. In recent years, tissue engineering, including the use of bioscaffolds, has demonstrated promising potential in bone repair [12,13,14]. Bioscaffolds provide a 3D microenvironment that promotes cell proliferation, migration, and differentiation at the defect site, thereby facilitating repair. However, the inherent lack of blood vessels and the limited proliferation capability of chondrocytes restrict the self-repair capabilities of cartilage [15,16]. Consequently, the introduction of external nutrients emerges as an effective strategy to accelerate the repair of articular cartilage. Similarly, providing essential nutrients for subchondral bone has also been recognized as vital approach to hasten the healing process.

Drug-loaded bioscaffolds can provide essential nutrients for tissue regeneration through the controlled release of encapsulated drugs. When integrated with the large-pore architecture of 3D-printed bioscaffolds, they further enhance cell adhesion and proliferation, thereby accelerating tissue regeneration. The initial drug-loaded bioscaffolds employed bare-metal stent platforms for the delivery of drugs targeting neointimal hyperplasia. These drugs were locally released within the vessel, effectively inhibiting intimal hyperplasia and thereby preventing in-stent restenosis. With advancements in research, the applications of drug-loaded bioscaffolds have expanded to other areas of tissue engineering. Recently, drug-loaded bioscaffolds are used for treating osteochondral defects, with their primary function being embedding drugs within the scaffold structure for precise and sustained release [17]. The principal aim of this scaffold design is to raise stem cells and promote their differentiation by regulating the kinetics of drug release. The current selection of drugs for drug-loaded bioscaffolds includes growth factors, ions, nanoparticles, anti-inflammatory drugs, and agents that promote cartilage repair [18,19,20,21,22,23]. Drug-loaded bioscaffolds exhibit higher bioactivity and superior therapeutic efficacy compared with conventional bioscaffolds [24]. Therefore, drug-loaded bioscaffolds are thus regarded as a viable therapeutic approach for osteochondral defects. The overview presented herein is timely due to recent innovations in the fabrication method of drug-loaded bioscaffolds, the diversity of drugs employed, and their broadening scope of applications. These evolving scenarios underscore the necessity for this comprehensive review. This article will highlight cutting-edge developments in drug-loaded bioscaffolds designed for cartilage and subchondral bone regeneration and discuss the latest insights into the pathological mechanisms of osteochondral defects. It is our hope that this review will establish a theoretical framework for the further development of drug-loaded bioscaffolds.

## 2. Osteochondral Defect and Its Clinical Treatment

### 2.1. Anatomy of Osteochondral Structure

**Figure 1:**
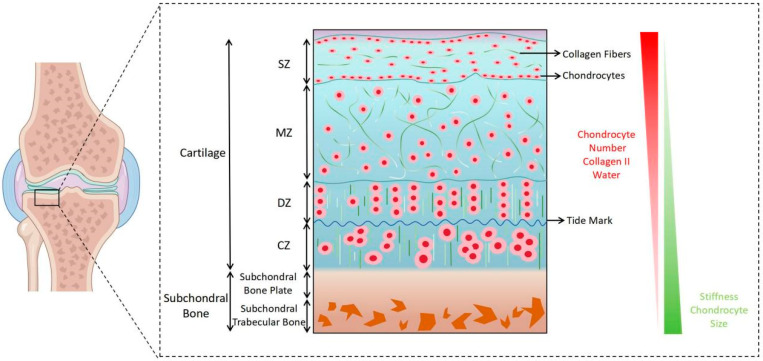
The schematic diagram of the microstructure and components of osteochondral tissue. Osteochondral tissue features a highly intricate structure, including cartilage, subchondral bone, and their transitional zone. From articular cartilage to subchondral bone, the chondrocyte numbers, collagen II, and water content gradually decrease, while the stiffness and chondrocyte size progressively increase. SZ: Superficial zone, MZ: middle zone, DZ: deep zone, CZ: calcified zone. By Figdraw (https://www.figdraw.com, accessed on 24 July 2024).

Osteochondral tissue features a highly intricate structure, including cartilage, subchondral bone, and their transitional zone (Figure 1). Different regions possess varying compositional ingredients, physiological structures, and biomechanical functions. The articular cartilage is a transparent layer of connective tissue located on the surface of active joints, which reduces the friction between adjacent bones [25]. It is primarily composed of the extracellular matrix (ECM) and chondrocytes. The ECM is composed primarily of water, collagen (mainly type II), and proteoglycans, with chondrocytes being the sole cellular components within articular cartilage. As the depth increases from shallow to deep, articular cartilage can be classified into four distinct zones: the superficial zone (SZ), middle zone (MZ), deep zone (DZ), and calcified zone (CZ) [26]. Each region exhibits distinct cell morphology and density, collagen fiber distribution, and ECM composition, catering to different biomechanical functions. The chondrocytes in the SZ are predominantly flat and feature a high concentration of collagen, which aids in reducing shear stress. In contrast, the DZ is characterized by spherical chondrocytes and a reduced collagen content, providing resistance to compressive forces [26]. The absence of nerves, blood vessels, and lymphatic vessels is a unique anatomical and physiological feature of articular cartilage, resulting in limited self-repair capability [27].

The subchondral bone, which is the deepest tissue within the osteochondral unit, comprises dense bone that provides biomechanical support and nutrition for articular cartilage [24,28]. Subchondral bone is primarily composed of hydroxyapatite (HA) and type I collagen, contributing to its significant stiffness and hardness [28]. Based on its structure and physiological characteristics, subchondral bone can be divided into two areas: the subchondral bone plate, which is closer to the calcified cartilage, and the trabecular bone, which is closer to the bone medullary cavity [26]. Unlike articular cartilage, which is characterized by a unique structure (no nerves, no blood vessels, and no lymphatic vessels), subchondral bone is highly vascularized, allowing for self-remodeling and regeneration. It is also well innervated, which may contribute to the pain associated with diseases such as osteoarthritis [25,26].

There is also a boundary layer structure between articular cartilage and subchondral bone: the cartilage–bone interface. Owning to its special location and concealment, the cartilage–bone interface has always been a focal point and challenge in the research of osteochondral unit. Anatomically, the bone–cartilage interface refers to the junction between articular cartilage and subchondral bone, serving as a juncture and connection hub between the two tissues [6,29]. Calcified cartilage is the primary component of the osteochondral interface, containing a significant amount of HA and collagen. The hardness of calcified cartilage is approximately 10 times that of hyaline cartilage and 1/60 that of bone [30], enabling it to mitigate mechanical impacts generated during joint movement and provide a protective effect on articular cartilage due to its special mechanical properties. In addition to calcified cartilage, the osteochondral interface features a wavy tidemark that distinguishes the deep layers of calcified and articular cartilage. Furthermore, the tidemark acts as a barrier, ensuring that only the arteries, veins, and nerves supplying the osteochondral unit can penetrate through to reach the calcified cartilage layer, thereby preventing further extension into the articular cartilage and serves to protect the hyaline cartilage [6].

### 2.2. The Progression and Pathological Features of Osteochondral Defects

Common causes of osteochondral defects include trauma and osteoarthritis. When trauma occurs, articular cartilage is the first area to be damaged, and with excessive mechanical stimulation, it can further destroy the subchondral bone. Studies have found that following an injury, chondrocytes at the edge of the injury gradually undergo apoptosis, resulting in a lower cell density of the cartilage tissue at the injured edge compared to in the healthy cartilage [31]. Chondrocytes, the only cellular component of articular cartilage, exhibit very low metabolic activity and virtually no regeneration capability, rendering damage that is often irreversible. Compared with trauma, the pathological process of osteochondral defects caused by osteoarthritis is more distinct. In the early stage of osteoarthritis, joint damage manifests only as abnormalities in the cartilage ECM [32]. Specifically, the excessive secretion of matrix metalloproteinases (mainly MMP13) by chondrocytes results in the excessive degradation of the ECM, and the net content of the cartilage ECM gradually decreases. The degradation of the ECM leads to excessive destruction of the cartilage collagen fiber network and causes the proteoglycans in the ECM to degrade into small-molecule structures, making it easier for them to escape from the collagen fiber network. This results in the loss of proteoglycans and undermines the collagen fiber network and microenvironment surrounding the chondrocytes. As the disease progresses, chondrocytes deprived of fibrous network bioscaffolds experience metabolic disorders under stress, resulting in aberrant differentiation and the generation of a substantial number of proliferative cluster chondrocytes and hypertrophic chondrocytes [33]. The alterations in the composition and structure of the articular cartilage further stimulate chondrocyte metabolic disorders, eventually leading to severe consequences such as cartilage structure damage, chondrocyte apoptosis, and damage or even loss of articular cartilage [34]. Following the stripping of articular cartilage, a series of lesions occur, including changes in the composition of subchondral bone and lamellar bone sclerosis. Osteophytes form at the edge of cartilage due to vascular proliferation. The synovial tissue of the joint becomes congested and edematous under the stimulation, leading to synovitis. Fibrosis of the proliferative synovial membrane causes joint capsule contracture, narrowing of the joint space, and other related diseases, which ultimately lead to significant damage to joint function and severe limitation of joint mobility [35].

### 2.3. Current Treatment Strategies for Osteochondral Defect

**Figure 2:**
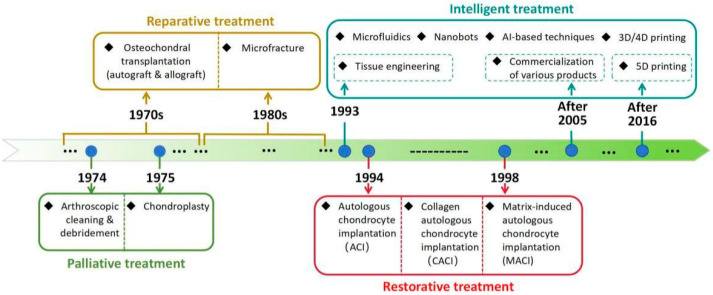
The development of osteochondral defect treatment methods. Current clinical strategies for osteochondral defects can be categorized as palliative treatments, reparative treatments, and restorative treatments. Future approaches will involve intelligent techniques such as tissue engineering, nanobots, 3D/4D/5D printing, AI-based techniques, and the commercialization of various products. 3D: three-dimensional, 4D: four-dimensional, 5D: five-dimensional, AI: artificial intelligence.

Currently, clinical treatment methods for osteochondral defects encompass both non-surgical and surgical treatments. The non-surgical treatment encompasses physical fixation, such as cast fixation, and drug therapy. Surgical treatment can be broadly classified into three types based on the treatment approach: palliative, reparative, and restorative treatments [6,25] (Figure 2). 

The specific methods of palliative treatment include arthroscopic cleaning, debridement, chondroplasty, etc. Among them, arthroscopic debridement is considered a minimally invasive procedure aimed at providing short-term symptom relief by reducing the inflammatory response and excising pathological structures [36]. The objective of chondroplasty is to create smooth edges using mechanical tools or radiofrequency energy without damaging the surrounding cartilage [25]. It can be seen that the primary goal of palliative treatment is to alleviate pain and enhance functional status, rather than replacing damaged tissue. Therefore, no matter which palliative method is used, it can only delay the disease process, but not prevent the progression of cartilage defects.

Unlike palliative treatment, which does not replace damaged tissue, reparative treatment replaces damaged or missing tissue by using additional biological materials [26]. Common reparative treatments include microfracture and autologous or allogeneic osteochondral transplantation. Microfracture, initially proposed in the 1980s, has quickly gained widespread acceptance among clinicians [37]. Its principle is to stimulate bleeding in the subchondral bone, the formation of blood clots, and the recruitment of bone marrow cells and other repair factors to form fibrocartilage, thereby facilitating the endogenous repair of both bone and cartilage [25]. This method has become the “classical method” in clinical treatment for osteochondral defects, and its short-term efficacy can be satisfactory [38]. However, microfracture can only generate fibrocartilage with low biomechanical and mechanical properties, thereby posing challenges in effectively replicating the functionality of natural articular cartilage [39,40]. In addition, the scope of applications for microfracture is limited, making it suitable only for early cases with a small defect area [41]. At present, autologous or allogeneic osteochondral transplantation is frequently employed in clinical treatment for the management of large-scale osteochondral defects [41]. The principle of both methods is to utilize healthy bone cartilage tissue or artificial bionic materials to repair the defect. The advantages of autologous cartilage transplantation lie in its ability to avoid immune reactions and effectively repair osteochondral defects. However, problems such as limited source of donors, mismatch of mechanical properties and secondary injury of autologous transplantation also impede the further advancement and application of this method [42,43]. The donor source for allogeneic osteochondral transplantation far exceeds that of autologous osteochondral transplantation. And compared with autologous osteochondral transplantation, allogeneic osteochondral transplantation can be applied to larger osteochondral defects. Studies have shown that autologous osteochondral transplantation is recommended for defects smaller than 1.5 cm^2^ with limited efficacy in bone marrow stimulation, whereas allogeneic osteochondral transplantation is advised for larger defects [44]. Despite the above advantages of allogeneic osteochondral transplantation, this method also entails risks such as difficulties in graft preservation and management, immunogenicity, and potential disease transmission, all of which may contribute to treatment failure [42,43].

Compared with palliative treatment and reparative treatment, restorative treatment appears to be more effective. The commonly used restorative treatments include ACI, collagen autologous chondrocyte implantation (CACI), and matrix-induced autologous chondrocyte implantation (MACI) [45,46]. Since its proposal in 1994, ACI remains the only clinical method utilizing tissue regeneration for the treatment of osteochondral defects [47]. The principle involves utilizing chondrocytes derived from healthy regions for in vitro cultivation, followed by their implantation into the defect site beneath the periosteal cover, thereby accomplishing osteochondral regeneration [25]. Studies have shown that 10 to 20 years after ACI, hyaline cartilage with almost the same mechanical strength and stable function can be regenerated at the transplant site. Moreover, the newly formed cartilage exhibits excellent integration with adjacent cartilage and subchondral bone [48,49]. After decades of development, ACI has undergone continuous improvement and innovation, evolving into MACI. This involves the isolation of autologous chondrocytes followed by in vitro culture using a matrix composed of porcine type I/III collagen. Subsequently, the cultured chondrocytes along with the matrix are transplanted into the targeted area [25]. This method can not only reduce the operation time, but also enhance chondrocyte proliferation, stabilize their phenotype, and make their distribution even [25]. However, compared with other single-operation surgeries, all generations of ACI necessitate a second operation, imposing physiological, psychological, and economic burdens on patients [50,51]. In addition, the application of ACI in osteochondral defects still faces the challenge of simultaneously inducing regeneration in the subchondral bone [52].

Although many of the above surgical methods have demonstrated efficacy in treating certain patients, each approach possesses inherent limitations. According to statistics, there are more than 2 million osteochondral transplantation operations performed worldwide annually, including many repeated operations and even suboptimal treatment outcomes [53]. Immune rejection, incision infection, and inadequate healing at the donor site may all be the reasons for surgical failure [54,55]. In addition, most of the current clinical strategies cannot achieve long-term effective treatment of the injured site and are unable to restore the original structure and function of osteochondral tissue [56]. Therefore, new methods are urgently needed to achieve both osteochondral injury repair and functional recovery. In recent decades, with the joint development of materials medicine and materials science, tissue engineering has emerged as a prominent approach in the field of osteochondral regeneration. Tissue engineering comprises three key components: cells, bioscaffolds, and growth factors [57,58]. Among them, bioscaffolds play a crucial role due to their unique 3D structure. Firstly, bioscaffolds can play a certain weight-bearing role; secondly, bioscaffolds can provide a microenvironment for various biological processes such as cell adhesion, migration, and proliferation. In short, bioscaffolds create conditions for the in situ regeneration and repair of osteochondral tissue [50,59]. In general, the design of a qualified bioscaffold should generally prioritize the following aspects: (1) The implanted bioscaffold exhibits excellent biocompatibility and demonstrates remarkable biodegradability. (2) The implanted bioscaffold possesses weight-bearing functionality and mechanical strength, thereby providing a more stable environment and framework for the regeneration of bone and cartilage. (3) The bioscaffolds, whether single-phase, biphase, three-phase, or polyphase scaffolds, should possess a specific pore size and porosity to facilitate cell proliferation and migration, nutrient transportation, and metabolic waste discharge [60,61]. To further improve the above properties, researchers have combined bioscaffolds with different types of drugs, offering a more efficient approach to osteochondral regeneration. Notably, many factors influence these properties, including preparation strategies and the types of loaded drugs, which we will focus on in the following sections.

## 3. Preparation Strategies of Drug-Loaded Bioscaffolds for Osteochondral Regeneration

Currently, methods for preparing bioscaffolds for osteochondral defects can be categorized into traditional techniques and contemporary techniques (Table 1). Traditional methods primarily include electrospinning, solvent casting/particle leaching, gas foaming, and phase separation. Although these traditional techniques can control certain key bioscaffold parameters to an extent, they fall short in biologically replicating the original microstructure of cartilage and subchondral bone [6]. In contrast, contemporary methods such as 3D/4D printing technology, which rely on advanced computer techniques, excel in fabricating tissue engineering bioscaffolds. These methods enable the creation of controllable and intricate microstructures tailored to meet specific requirements [62,63].

### 3.1. Traditional Methods

Electrospinning is a process in which a polymer solution, emulsion, or melt is extruded through a spinneret under the influence of a strong electric field, resulting in the production of fibers that are deposited on an appropriate collector [64]. This method not only offers cost-effectiveness, versatility, and simplicity of operation but also enables compatibility with a wide range of polymers for forming fiber webs with varying chemical compositions and associated properties [64,65]. However, the bioscaffolds formed through electrospinning exhibit a small pore size, which may impede cell migration, nutrient cycling, and waste metabolism [66,67]. Additionally, electrospinning often requires the utilization of organic solvents, which can be toxic to cells and may pose environmental risks. 

Solvent casting/particle leaching requires dissolving the bioscaffold materials in a corresponding solvent and then evenly dispersing a water-soluble porogen of the selected size into the solution to form a polymer–porogen complex [68]. This technique is simple and widely applicable [69], allowing for easy independent adjustment of porosity and pore size. However, similarly to electrospinning, it often results in limited mechanical properties of the synthesized bioscaffolds due to the use of organic solvents during pore formation [70].

Gas foaming utilizes the principle of varying gas solubility in solids under different pressures. By altering the pressure, gas cavities are formed within the polymer, ultimately resulting in a porous scaffold [70,71]. Gas foaming can be classified into physical and chemical foaming [72]. Physical foaming, which avoids the use of organic solvents, is considered more environmentally friendly. However, regardless of the foaming method, achieving precise control over the pore size of scaffolds synthesized via gas foaming remains a challenge, often resulting in internal pore blockages within the polymer structure [73]. To address this issue effectively, it is possible to enhance the process by combining gas foaming with particle leaching [74]. Furthermore, poor inter-pore connectivity is another limitation for bioscaffolds produced through gas foaming.

Phase separation methods are classified into two categories: thermally induced phase separation (TIPS) and nonsolvent-induced phase separation (NIPS) [75]. TIPS, commonly known as the freeze-drying method, employs low temperatures to induce phase separation, while NIPS is facilitated by the addition of a nonsolvent [76]. Due to its ability to create high porosity without requiring high-temperature conditions, the phase separation method has attracted significant research interest. However, bioscaffolds synthesized using this approach often have the issue of small pore size, making it difficult to control the macro- and microstructure of the material. In addition to these four common methods, melt molding [77] and selective enzymatic degradation [78] are also traditional approaches. Nevertheless, none of these methods fully satisfy the requirements of researchers and clinicians.

### 3.2. Contemporary Methods

Contemporary methods for fabricating osteochondral regenerative bioscaffolds are centered around additive manufacturing techniques, such as 3D printing, 4D printing, and 5D printing. 

Three-dimensional printing, also known as additive manufacturing (AM), utilizes computer-aided design (CAD) and computed tomography (CT) images. Since 1986, 3D printing has undergone nearly 40 years of development and has been applied to numerous fields, including bone tissue engineering [79]. It offers advantages in creating porous and customized scaffold designs [80,81]. The 3D printing process involves multiple devices working together in tandem: first, a model is built from CAD or CT images, then imported into a computer connected to the printing device, and finally printed using bioink. Based on the different 3D printing processes, common 3D printing technologies primarily include the following: (1) Material extrusion (e.g., fused deposition modeling (FDM), extrusion-based bioprinting) [82]. Material extrusion is currently the most commonly used 3D printing technique [82]. The technique is applicable to a wide range of materials, including polycaprolactone, gelatin, hyaluronic acid, calcium silicate/strontium phosphate (CaSiO_3_/Sr_3_(PO_4_)_2_), and others [83,84,85,86,87]. Therefore, the selection of materials can vary based on specific requirements and application contexts. For example, the polymer-based material extrusion technique can print porous materials most similarly to subchondral bone, while the bioceramic-based material extrusion technique is mainly used for calcified cartilage and the subchondral bone layer of osteochondral bioscaffolds [83,88]. Compared with other methods, material extrusion offers several advantages including simple equipment, a wide range of material options, and the ability to create relatively porous high-porosity bioscaffolds. However, it is important to note that this technique faces challenges such as low printing accuracy, long printing time, and a lack of standardization in the printing process [89,90]. (2) Powder bed fusion (e.g., selective laser sintering (SLS)) [82]. The materials utilized in this technique for bone applications encompass poly(ε-caprolactone) (PCL), biphase calcium phosphate, titanium alloys, and other related substances [91,92,93]. The primary benefit of this technique lies in its capacity to manufacture exceptionally refined and robust porous bioscaffolds [94]. However, the sintering process of the scaffold generates local ultra-high temperatures, which renders it impossible to incorporate cells, proteins, or thermally unstable bioactive molecules simultaneously [94]. (3) Vat photopolymerization (e.g., stereolithography (SLA) and digital light processing (DLP)) [82]. The base material used in this technique is compatible with many of the above materials, but generally requires extensive modification of the material [95]. At present, the common scaffold materials for osteochondral repair using this technique encompass poly (ethylene glycol) (PEG), gelatin methacryloyl (GelMA), and tricalcium phosphate (TCP) [83,96,97]. The combination of high precision and efficient manufacturing makes vat photopolymerization a highly promising technique for 3D printing. However, compared with other technologies, the scaffold printed by this technique does not possess any porosity advantage [83]. In addition, the limitations of printing technology, restricted material selection, and substantial upfront investment and maintenance costs have become the main challenges facing this technique [98]. (4) Inkjet bioprinting (including thermal inkjets, piezoelectric inkjets, and electrostatic inkjets) [99]. This technique employed in the process utilizes liquid droplets as its fundamental building blocks, which are precisely dispensed onto the substrate through on-demand printing to create 3D structures [99]. Compared with material extrusion, inkjet bioprinting has small droplets and a high resolution [100]. In addition, its simplicity and flexibility make this technique widely used. However, the narrow application range of materials and the difficulty in printing complex 3D structures still limit its further application [100].

Three-dimensional printing can be regarded as a groundbreaking technological revolution in the realm of contemporary medicine [101,102]. Many existing 3D printing technologies can also be used for the preparation of osteochondral drug-loaded bioscaffolds. In terms of material selection, currently commonly used printing materials include polymers (such as PCL, polyvinyl alcohol (PVA) and hydrogels), metals (such as titanium (Ti), magnesium (Mg)) and bioceramics (such as TCP, bioactive glass (BG)). In general, hydrogels are primarily utilized for printing hyaline cartilage layers due to their hydration and viscoelastic properties closely resembling those of natural ECMs. Bioceramics, hyaluronic acid, TCP, and metallic materials are better suited for printing subchondral bone [1,103]. Therefore, future endeavors will focus on enhancing the biocompatibility and plasticity of printing materials.

In general, compared to traditional methods, 3D printing technology enables independent regulation of the macro- and micro characteristics of the bioscaffold and can closely replicate the anisotropy of the ECM and the heterogeneity of osteochondral tissue. Therefore, bioscaffolds fabricated by 3D printing are more suitable for bone and cartilage tissue regeneration. Moreover, 3D printing technology can provide personalized printing methods and treatment options to achieve precision medicine. However, the limited range of materials suitable for 3D printing, along with the time and cost associated with layer-by-layer processing, are disadvantages of this technology. These limitations also hinder further development of the technology and the realization of large-scale and industrial production [104,105]. Human tissue undergoes a dynamic process of healing and regeneration, yet bioscaffolds produced by traditional methods or 3D printing possess only static properties and behaviors, which do not align with the dynamic evolution of human tissue or fulfill biomedical requirements. 

Four-dimensional printing technology is the introduction of a fourth dimension—time—to three-dimensional printing technology, enabling the print material to react and adapt to a variety of stimuli such as temperature, water, pH, and light [106]. In orthopedics, 4D-printed bioscaffolds can more accurately conform to the geometry of the bone defect area over time, and their functional transformation during the post-printing stage can synchronize with the natural healing mechanism and promote dynamic bone remodeling [107,108,109]. To date, there have been reports of 4D printing for bone repair [110]; however, its application in cartilage repair and osteochondral defect repair remains scarce, indicating a need for further clinical exploration and trials in this area. Currently, 4D printing is still in the research stage. Before it can be applied clinically, numerous challenges must be addressed [111]. For instance, developing suitable biomaterials is challenging, because smart materials that respond to stimuli may struggle to balance good biocompatibility and mechanical properties. Moreover, most bioscaffolds can only respond to a single stimulus, whereas the human body frequently encounters multiple stimuli simultaneously. Furthermore, the precise biological interaction mechanisms between material and bone still remain unclear at this stage. Addressing these issues in the future may enhance the efficiency and outcome of 4D printing and further promote the development of 4D printing technology in bone-related fields. Interestingly, the emergence of 5D printing technology has expanded the capabilities of 4D printing by incorporating information as the fifth dimension into printed structures, thereby enabling it to encompass curved layers [112]. 

Five-dimensional printing technology is a new concept. Although currently speculative, 5D printing surpasses traditional additive manufacturing methods like 3D printing and 4D printing. Though lacking a universally acknowledged definition, 5D printing usually integrates extra dimensions beyond the three spatial dimensions (length, width, height) and the fourth dimension of time. This can potentially include aspects such as material composition, structure, or functionality that change over time or respond to external stimuli. It represents a speculative concept delving into the potential future trajectory of additive manufacturing technology. Therefore, the future development of 5D printing in osteochondral defect treatment is also promising [113].

**Table 1:** Summary of preparation strategies of drug-loaded bioscaffolds.

Preparation Strategies	Technologies		Advantages	Disadvantages	Ref.
Traditional methods	Electrospinning		(1) Cost-effectiveness. (2) Versatility. (3) Easy to operate.	(1) Small pore size of the bioscaffold. (2) Use of organic solvents.	[64,65,66,67]
	Solvent casting/particle leaching		(1) Easy to operate. (2) Easy to adjust the pore size and porosity of the bioscaffold.	(1) Use of organic solvents. (2) Limited mechanical properties of bioscaffolds.	[69,70]
	Gas foaming	(1) Physical foaming (2) Chemical foaming	Environmentally friendly (physical foaming)	(1) The pore size of bioscaffolds is uncontrollable. (2) Non-connected pores. (3) Poor inter-pore connectivity of the bioscaffold.	[72,73]
	Phase separation	(1) TIPS (2) NIPS	(1) High porosity of the bioscaffold. (2) Does not require high-temperature conditions.	(1) Small pore size of the bioscaffold. (2) Difficult to control the macro- and microstructure of the bioscaffold.	[75,76]
Contemporary methods	3D printing	(1) Material extrusion (2) Powder bed fusion (3) Vat photopolymerization (4) Inkjet bioprinting	(1) More suitable for bone and cartilage regeneration. (2) Precision treatment. (3) Personalized customization.	(1) Limited material categories. (2) Long synthesis time. (3) High cost.	[104,105]
	4D printing		(1) Stimulus responsiveness. (2) More suitable for defects.	Still in research.	[106,107,110,111,112]
	5D printing		Changes in material composition, structure, or functionality.	Still in concept.	[113]

TIPS: thermally induced phase separation; NIPS: nonsolvent-induced phase separation; 3D: three-dimensional, 4D: four-dimensional, 5D: five-dimensional.

## 4. Classification of Drugs Loaded in Bioscaffolds

**Figure 3:**
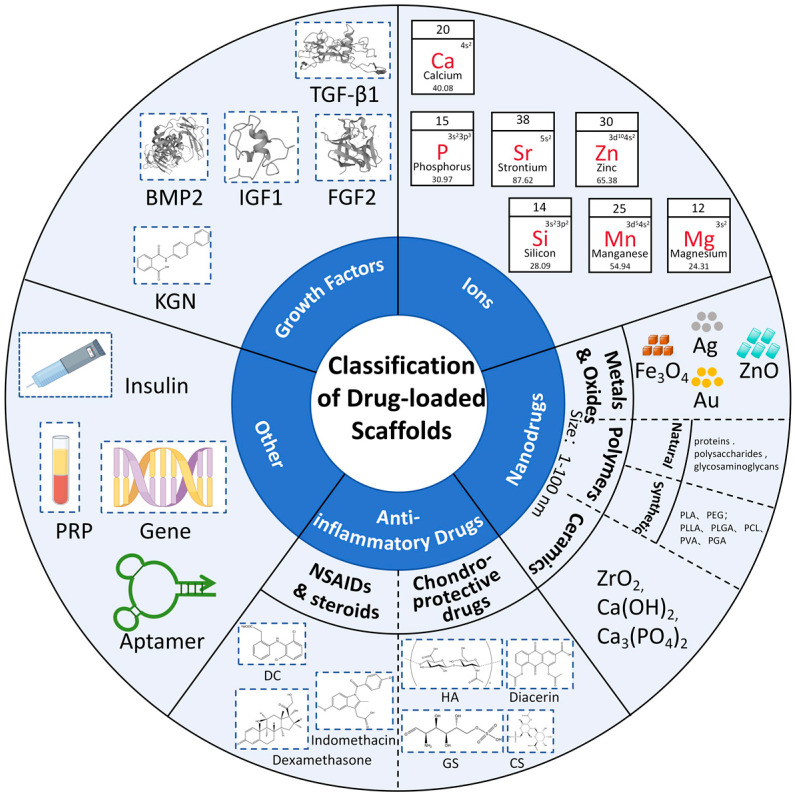
Specific classification of the drugs encapsulated within bioscaffolds. The primary drugs encapsulated within drug-loaded bioscaffolds include growth factors, nanoparticles, ions, anti-inflammatory drugs, and other pharmaceutical agents. The representative drugs under different categories are listed. TGF-β1: transforming growth factor-β1, BMP2: bone morphogenetic proteins 2, IGF1: insulin-like growth factors 1, FGF2: fibroblast growth factor 2, KGN: kartogenin, Ca: calcium, P: phosphorus, Sr: strontium, Zn: zinc, Si: silicon, Mn: manganese, Mg: magnesium, Ag: silver, Au: gold, Fe_3_O_4_: iron(II,III) oxide (also known as magnetite), ZnO: zincoxide, PLA: polylactic acid, PEG: poly (ethylene glycol), PLLA: poly(L-lactic acid), PLGA: poly(lactic-co-glycolic acid), PCL: poly(ε-caprolactone), PVA: poly(vinyl alcohol), PGA: poly(glycolic acid), ZrO_2_: zirconium dioxide, Ca(OH)_2_: calcium hydroxide, Ca_3_(PO_4_)_2_: calcium phosphate tribasic, DC: diclofenac sodium, HA: hyaluronic acid, GS: glucosamine sulfate, CS: chondroitin sulfate, PRP: platelet-rich plasma, NSAIDs: nonsteroidal anti-inflammatory drugs. By Figdraw (https://www.figdraw.com, accessed on 24 July 2024).

Drug-loaded bioscaffolds, which possess drug-carrying capabilities, are extensively utilized in the field of osteochondral repair [114]. Currently, the primary drugs encapsulated within drug-loaded bioscaffolds include growth factors, nanoparticles, ions, anti-inflammatory drugs, and other pharmaceutical agents (Figure 3). 

**Figure 4:**
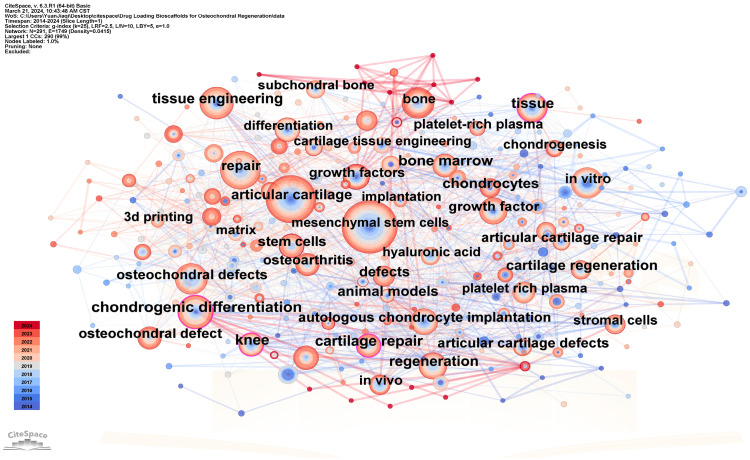
Bibliometric analysis of the literature published within the last decade on drug-loaded bioscaffolds for osteochondral defect regeneration. The keyword co-occurrence mapping shows that N = 291, E = 1749, indicating that there are 291 keywords that appear and 1749 times that the keywords are related to each other.

To provide a comprehensive overview of the research landscape concerning bioscaffolds, we employed CiteSpace v.6.3.R1 software to conduct a bibliometric analysis of the literature published within the last decade in the relevant field. The publications were sourced from the Web of Science Core Collection database (WoSCC). The drugs loaded on the bioscaffolds were classified by categorizing and designing keywords. Our search strategy involved a Topic search (TS) #1 = (“growth factors”) OR (“nanoparticles”) OR (“anti-inflammatory drugs”) OR (“ions”) OR (“aptamers”) OR (“PRP”) OR (“insulin”) AND TS #2 = (“scaffold”) AND TS #3 = (“osteochondral defect”). Following data cleaning, duplicate publications were eliminated using CiteSpace v.6.3.R1. Ultimately, 466 unique records were included in the final analysis. Subsequently, all records were imported into CiteSpace v.6.3.R1 (Figure 4). Out of the 466 keywords analyzed, those with a frequency exceeding 40 included “mesenchymal stem cells”, “articular cartilage”, “repair”, “chondrogenic differentiation”, “osteochondral defects”, “regeneration”, “tissue engineering”, “in vitro”, “bone”, “tissue”, and “growth factor”. The types of growth factors loaded on bioscaffolds and their applications are displayed in Table 2. 

**Figure 5:**
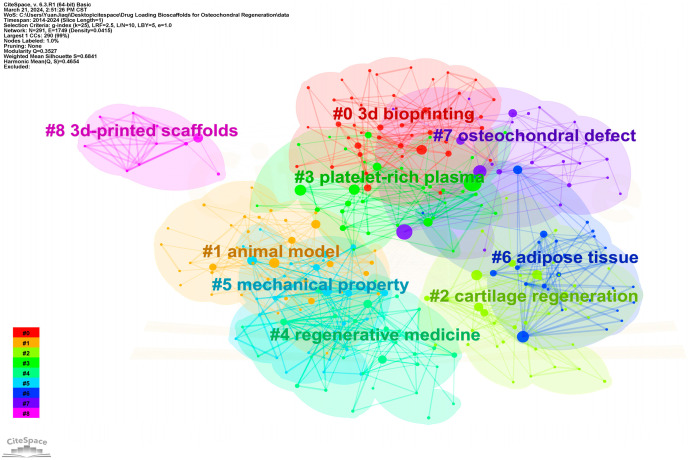
Keyword clustering mapping of drug-loaded bioscaffolds for osteochondral defect regeneration based on CiteSpace v.6.3.R1. Modularity (Q) = 0.3527, silhouette (S) = 0.6841. Q is an index for evaluating the modular structure of the network, with a value interval of [0, 1], and the obtained modular structure of the network is considered significant when Q > 0.3. S is an index for evaluating the homogeneity of the network, and the closer S is to 1, the higher the homogeneity of the network is; when S > 0.5, the clustering results are considered reasonable.

These keywords underscore that, concerning osteochondral defects, researchers are primarily investigating the in vitro osteogenic and chondrogenic differentiation of mesenchymal stem cells (MSCs) using drug-loaded bioscaffolds, particularly those loaded with growth factors. Conversely, researchers are also establishing animal models of osteochondral injury and subsequently implanted drug-loaded bioscaffolds in vivo to promote the regeneration of articular cartilage and bone. Furthermore, we conducted a cluster analysis of the keywords, revealing clear and reasonable clustering patterns. As illustrated in Figure 5, nine distinct clusters emerged, namely “3D bioprinting”, “animal model”, “cartilage regeneration”, “platelet-rich plasma”, “regenerative medicine”, “mechanical property”, “adipose tissue”, “osteochondral defect”, and “3D-printed scaffolds”. Similarly to the keyword mapping results, the clustering outcome suggest that drugs loaded on 3D-printed bioscaffolds predominantly consist of regenerative agents, while the scaffold primarily serves to offer mechanical support. Consequently, the mechanical properties of the bioscaffolds assume particular significance. Below, we will further categorize the drugs loaded on the bioscaffolds. 

### 4.1. Growth Factor-Loaded Bioscaffolds

Growth factors are a class of biologically active molecules, usually proteins or peptides, that regulate cellular physiological functions in organisms by attaching to cell surface receptors and triggering intracellular signaling pathways. These factors are crucial in biological processes like cell proliferation, differentiation, migration, and programmed cell death [115]. However, utilizing simple biochemical factors for the long-term treatment of osteochondral defects is challenging due to their short duration of action, difficulty in dose control, lack of local concentration advantage in systemic administration, and insufficient spatial support [6]. As an advanced treatment method, bioscaffolds combined with biochemical factors have been widely employed in repairing osteochondral defects. The scaffold plays a vital supporting and guiding role in this process, enabling the sustained and controlled release of biochemical factors, improving local drug concentration, and avoiding the systemic side effects of drug delivery. To accomplish the loading and regulated release of biochemical factors, the selection of bioscaffolds is crucial. Bioscaffolds should be biocompatible, mechanically strong, and structurally stable to provide an ideal microenvironment. Additionally, the selection and release strategies of biochemical factors are critical for treatment success. Currently, the primary biochemical factors used in treating osteochondral defects include transforming growth factor-β (TGF-β), bone morphogenetic proteins (BMPs), insulin-like growth factors (IGFs), fibroblast growth factors (FGFs), and platelet-derived growth factor (PDGF) (Table 3).

The transforming growth factor superfamily comprises TGF-βs, activins, BMPs, and other related proteins. TGF-β1, a member of the transforming growth factor family, promotes chondrogenesis, subchondral osteogenesis, and wound healing, playing a crucial role in preserving cartilage integrity and attenuating cartilage degradation [116,117,118]. To improve the osteochondral regenerative effect, TGF-β1 was combined with stromal cell-derived factor 1-α (SDF-1α) to fabricate a novel sustained slow-release bioscaffold [18]. The results demonstrated that the TGF-β1 released from bioscaffolds could promote MSC homing, migration, and chondrogenic differentiation, and facilitate the osteochondral regeneration of rat knee joints. However, the short half-life and rapid metabolism of TGF-β1 have limited its clinical application. To enhance the drug circulatory time, a functionalized peptide hydrogel which combined TGF-β1 mimetic peptides with self-assembling peptides was developed. The results indicated that the hydrogel could significantly increase the expression of chondrogenic genes and ECM deposition [119,120]. 

BMPs (e.g., BMP-2, 4, 5, 6, 7, and 9), which belong to the TGF-β superfamily, have demonstrated considerable osteogenic activity [121]. Among them, BMP-2 and BMP-7 are more widely used. They can induce chondrogenic differentiation of MSCs, and BMP-7 also can promote the osteogenic differentiation of MSCs [122,123], while the absence of BMP-7 leads to the degeneration of articular cartilage and synovial inflammation during the developmental process [124]. Research has found that tantalum scaffolds loaded with BMP-7 achieved higher histological scores of osteochondral repair [125]. 

In addition to TGFs, FGFs and IGFs play pivotal roles in osteochondral repair. FGFs, especially FGF-2, are found in normal cartilage, which can stimulate chondrocytes to synthesize the cartilage matrix [126]. It was reported that porous hydroxyapatite/collagen bioscaffolds loaded with FGF-2 could significantly promote the synthesis of cartilage matrices and stimulate osteochondral regeneration [127]. Moreover, it has also been observed that exogenous basic FGF (bFGF) upregulates the expression of several cytokines to treat osteochondral defects in rabbit models [128].

IGF-1 serves both paracrine and autocrine functions and is the primary anabolic growth factor in articular cartilage, playing a crucial role in bone and cartilage repair [129]. Lu et al. engineered various bilayered, biodegradable hydrogels loaded with IGF-1 and BMP-2. They discovered that the concurrent delivery of IGF-1 and BMP-2 resulted in a higher percentage of subchondral bone regeneration, increased bone growth at the defected edge, and a lower specific surface area of the bone, yielding better outcomes compared to the administration of IGF-1 alone [130].

However, some researchers have found that while the scaffold delivery of IGF-1 alone demonstrated significant repair effects on rabbit osteochondral cartilage, its effectiveness was not sustained when co-loaded with TGF-β1 and implanted into the defect site. This raises an important question: could the synergistic delivery of different growth factors result in diminished or even lost efficacy [131]?

**Table 3:** Types of growth factors loaded on scaffolds and their applications.

Growth Factors	Subtype	Scaffolds	Applications	Ref.
TGF-β	TGF-β1	Poly (Ethylene Glycol)-Block-Poly(ε-Caprolactone)	Osteochondralregeneration	[132]
	TGF-β2	Biphasic silk fibroin scaffolds	Enthesis repair	[133]
	TGF-β3	Poly (Vinyl Alcohol)-Chitosan-Poly (Ethylene Glycol)	Chondrogenic differentiation of MSC	[134]
BMP	BMP-2	Silk Fibroin Scaffold	Bone regeneration	[135]
	BMP-4	Collagen gel/Fibrin sealant/Gelatin sponge carriers	Bone regeneration	[136]
	BMP-6	MOF-embedded electrospun fiber scaffold	Bone regeneration	[137]
	BMP-7	Polydopamine/Poly(lactic-co-glycolic acid)/hydroxyapatite	Calvarial repair	[138]
	BMP-9	Polylactic acid glycolic acid scaffold	Bone repair	[139]
IGF	IGF-1	Poly(lactic-co-glycolic acid)	Osteochondral regeneration	[140]
FGF	FGF-2	nHAP-coated PCL/HAP/beta-TCP scaffold	Bone regeneration	[141]
PDGF	PDGF-BB	Hydroxyapatite (HAp) and silk fibroin (SF) scaffolds	Osteochondral regeneration	[142]

TGF: transforming growth factor, BMP: bone morphogenetic protein, FGF: fibroblast growth factor, IGF: insulin-like growth factor, PDGF: platelet-derived growth factor.

In summary, the growth factors released by bioscaffolds can enhance the osteochondral repair performance by promoting MSC homing, inducing cell proliferation, and regulating MSC differentiation. 

### 4.2. Ion-Loaded Bioscaffolds

**Figure 6:**
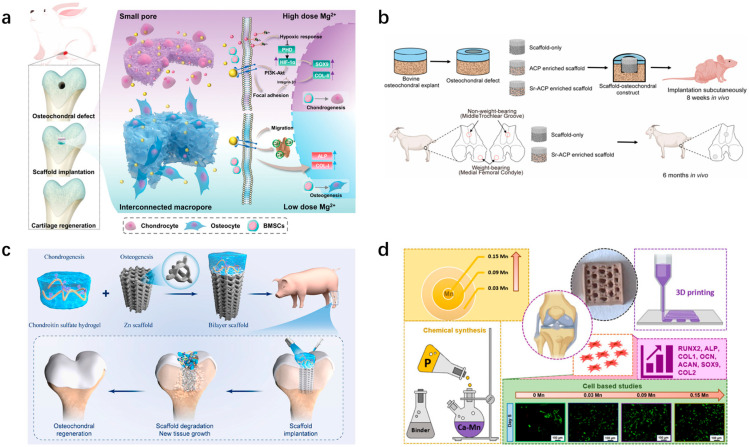
Common ions loaded on bioscaffolds designed for the treatment of osteochondral defects. (**a**) A double-layered scaffold, denoted as D-Mg-DFO, was prepared to incorporate the gradient release of Mg along with DFO. This scaffold regulates the chondrogenic and the osteogenic differentiation of BMSCs, thereby achieving optimal regeneration for osteochondral defects. (Reprinted with permission from Ref. [23]. Copyright 2023 John Wiley & Sons Books.) (**b**) Scaffold-loaded amorphous calcium phosphate particles (100–150 μm) enriched with Sr ions for treating osteochondral defects in mice and goats. (Reprinted from Ref. [148].) (**c**) A biodegradable bilayer scaffold consists of a CS hydrogel for regenerating cartilage tissue and a porous pure Zn scaffold for the regeneration of the underlying bone tissue. (Reprinted from Ref. [154].) (**d**) Multifunctional unit bioscaffolds with different manganese concentrations were prepared by 3D printing to promote osteochondral regeneration through endochondral differentiation. (Reprinted with permission from Ref. [157]. Copyright 2022 American Chemical Society.) BMSCs: bone marrow mesenchymal stem cells; Sr: strontium; CS: chondroitin sulfate; Zn: zinc.

Among the ions commonly involved in osteochondral repair are calcium (Ca), phosphorus (P), strontium (Sr), zinc (Zn), silicon (Si), manganese (Mn), and magnesium (Mg) [143]. These ions are crucial in bone biology and cartilage tissue engineering, promoting chondrocyte proliferation, differentiation, and matrix synthesis (Figure 6). Ca and P are the primary inorganic components of bone, essential for bone growth and the maintenance of hardness. Mg has been shown to accelerate cell migration, enhance angiogenesis, and promote the regeneration of vascularized bone tissue [144]. Researchers have also explored the application of bioscaffolds loaded with Mg for the treatment of osteochondral defects, designing a bilayer scaffold consisting of a hydrogel layer on top of a porous cryogel, designed to mimic the layered structure of osteochondral tissue. Mg was incorporated into the bilayer scaffold, significantly promoting the bilinear regeneration of cartilage and subchondral bone. Higher Mg concentrations in the top hydrogel layer enhanced chondrogenic differentiation. The bottom cryogel, supplemented with lower Mg, featured an interconnected macroporous structure supporting various functions, including the migration of MSCs from the bone marrow cavity, matrix mineralization, and osteogenesis [23]. Sr is an osteophilic element that enhances the osteoconductive properties of calcium phosphate and improves the mechanical properties of bone tissue [145]. As a trace element closely related to calcium, Sr can promote osteoblast differentiation while inhibiting osteoclast resorption [146,147]. Sr can be co-loaded onto bioscaffolds with a variety of ions to exert synergistic effects and promote osteochondral regeneration. For instance, researchers have demonstrated that incorporating Sr-rich amorphous calcium phosphate particles into collagen/Mg-hydroxyapatite osteochondral bioscaffolds improved subchondral bone repair [148,149]. Additionally, some researchers have constructed Sr_5_(PO_4_)_2_SiO_4_ (SPS) bioscaffolds through 3D printing using bioceramics releasing Sr and Si, which can aid in repairing osteochondral defects and effectively rebuild the intricate interface between cartilage and subchondral bone [150]. Zn can exhibit antioxidant and anti-inflammatory properties through the regulation of free radical formation in the body [151]. Compared to Mg-based materials, Zn-based materials exhibit superior biodegradation properties as they do not generate hydrogen gas or cause significant pH fluctuations [152]. Researchers have used Zn–cobalt bimetallic organic-framework-modified bioceramic bioscaffolds for repairing osteochondral defects within an osteoarthritis environment. The findings suggested that the bioscaffold accelerated the combined regeneration of cartilage and subchondral bone in cases of severe osteochondral defects [153]. Yang et al. designed a biodegradable bilayer scaffold that includes a chondroitin sulfate (CS) hydrogel for the regeneration of cartilaginous tissues and a pure zinc porous scaffold for the regeneration of subchondral bone to provide mechanical support for the cartilaginous layer [154]. They demonstrated in swine that this approach could significantly facilitate osteochondral regeneration. Furthermore, Li has been shown to be effective in arthritis treatment by activating the Wnt signaling pathway and promoting cartilage regeneration [155]. Studies have demonstrated that incorporating Li into calcium silicate bioscaffolds enhanced the secretion of osteochondral-related regeneration factors and promoted osteochondral regeneration [156]. Furthermore, Mn plays an important role in osteochondral repair. Some researchers have found that scaffolds prepared using Mn-doped calcium-deficient apatite can promote osteochondral repair [157]. In addition to the metal ions mentioned above, Si and P, as essential macronutrients, positively affect blood vessel formation and collagen deposition [158,159]. Previously, researchers have used two-dimensional P–silicon nanosheets to form a hydrogel scaffold, and they have found that this scaffold can achieve the controlled release of elemental Si and P to promote angiogenesis and osteogenesis [160]. Furthermore, they are involved in bone formation and cartilage synthesis processes and promote collagen synthesis, which is essential for the structure and strength of cartilage.

In osteochondral repair, ions play a crucial role in bone biology and cartilage tissue engineering by promoting osteoblast proliferation, differentiation, and matrix synthesis. Loading these ions into bioscaffold materials enhances their bioactivity and functionality, aiding osteochondral repair. Research in this area is vital for developing more efficient bioscaffolds.

### 4.3. Nanodrug-Loaded Bioscaffolds

Nanodrugs, also known as nanomedicines or nanoparticle drugs, are drugs or therapeutic agents that use nanotechnology to deliver, target, or enhance therapeutic effects. These drugs typically consist of nanoscale structures or nanodrugs engineered to interact with specific biological targets at the molecular level [161,162,163]. Nanoparticle drugs for osteochondral repair typically consist of minute particles with diameters ranging from 1 to 100 nm, and their types span a broad spectrum of materials such as metals, ceramics, and polymers (Table 4). Metallic nanodrugs, such as iron, silver, gold, and copper, exhibit good electrical conductivity and biocompatibility [164,165,166,167]. Polymer nanodrugs, comprising natural polymers (proteins, polysaccharides, glycosaminoglycans) and synthetic polymers, like polylactic acid (PLA), poly (ethylene glycol) (PEG), poly (propylene glycol) (PPG), and their derivatives, poly (L-lactic acid) (PLLA) and poly (lactic-co-glycolic acid) (PLGA), poly(ε-caprolactone) (PCL), poly (vinyl alcohol) (PVA), and poly (glycolic acid) (PGA) [168], feature tunable physical and chemical properties [169]. Ceramic nanodrugs, including zirconium oxide, calcium hydroxide, and calcium phosphate, possess high biocompatibility and bioactivity, and can enhance the mechanical strength of bioscaffolds [170]. 

The nanomedicines mentioned above can be internalized by cells, and improve the microenvironment of the damaged area, thereby treating osteochondral defects. Compared to conventional bioscaffolds, nanodrugs offer a large surface area and can establish a tight interface with the polymer matrix, improving mechanical properties while preserving excellent bone specificity and biocompatibility. This, in turn, affects protein adsorption, cell proliferation, adhesion, and differentiation, leading to the formation of new tissues [192]. It is noteworthy that the current study has discovered that, apart from remarkable outcomes in enhancing the mechanical properties of the bioscaffolds, nanodrugs can also induce certain distinctive biological effects, thereby facilitating the repair process at the damaged site. For example, Deng et al. reported that hair-derived antioxidative nanodrugs can help scavenge reactive oxygen species (ROS) [193]. Moreover, some nanodrugs with magnetic properties, such as iron oxide nanodrugs, can be coupled with an external field to generate magneto-mechanical forces to promote osteochondral regeneration [194,195].

### 4.4. Anti-Inflammatory Drug-Loaded Bioscaffolds

The overexpression of inflammatory factors (interleukin-1 beta (IL-1β), tumor necrosis factor-α (TNF-α), and cytokines like interleukin-6 (IL-6), interleukin-8 (IL-8), interleukin-17 (IL-17), and interleukin-18 (IL-18)) is the primary cause of osteochondral damage. The function of various cell types (such as osteocytes, chondrocytes, immune cells, and fibroblasts) in osteochondral sites is influenced by inflammatory factors via the relevant signaling pathways, ultimately resulting in further deterioration of the microenvironment in osteochondral defects [196], and the local inflammatory microenvironment not only degrades and metabolizes the ECM in articular cartilage, but also induces the apoptosis of chondrocytes [197,198,199,200,201,202,203,204,205]. Therefore, anti-inflammatory drugs are considered an effective therapy for osteochondral defects.

**Figure 7:**
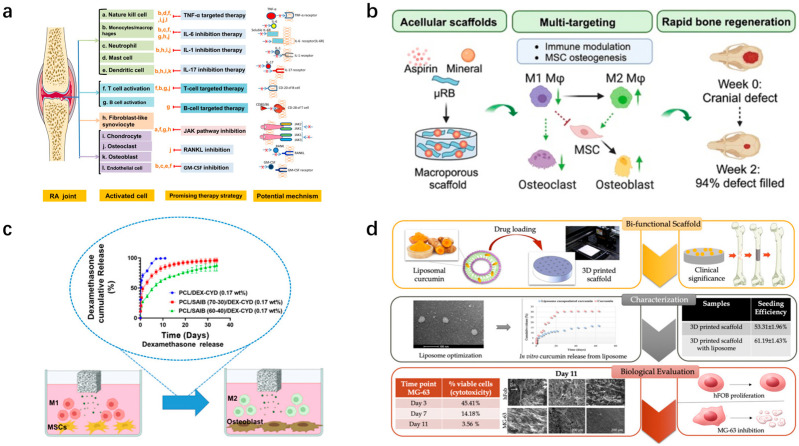
Bioscaffolds loaded with anti-inflammatory drugs for treating osteochondral defects, along with elucidation of underlying mechanisms. (**a**) Cell types and signaling pathways involved in osteochondral damage due to RA. (Reprinted from Ref. [196]). (**b**) Investigators coated mineral particles and aspirin on gelatin μRB supports. (Reprinted from Ref. [206].) (**c**) Dexamethasone-loaded bioscaffolds promote macrophage phenotypic shift from M1 to M2 and promote osteogenic differentiation of MSC. (Reprinted from Ref. [213]). (**d**) Curcumin-loaded 3D bioscaffolds promote cartilage matrix formation. (Reprinted from Ref. [216].) RA: rheumatoid arthritis; μRB: microribbon.

The primary anti-inflammatory drugs employed for osteochondral injuries include nonsteroidal anti-inflammatory drugs (NSAIDs) and steroids. Aspirin, a widely used NSAID, has been studied extensively. In in vivo experiments, micron-sized tricalcium phosphate (mTCP)-coated µRB bioscaffolds containing 20 µg of aspirin were found to result in the nearly complete healing of critical-sized cranial defects by 2 weeks [206] (Figure 7b). Among NSAIDs, diclofenac sodium is also commonly used. It has reported that prepared porous sintered bioscaffolds consisting of PLGA and PEG containing diclofenac sodium is suitable for treating acute inflammation, with approximately 80% of the drug being released within the initial 4 days, followed by a daily release rate of approximately 0.2% [207]. Similarly, indomethacin loaded onto bioscaffolds has been found to reduce lipopolysaccharide (LPS)-induced secretion of pro-inflammatory factors from macrophages, modulate the degradation of the ECM by chondrocytes, and promote the formation of new collagen by osteoarthritic chondrocytes [208]. In addition, steroids can also be loaded onto bioscaffolds for the treatment of osteochondral defects. The main steroid drugs commonly used for osteochondral repair include dexamethasone, prednisolone, methylprednisolone, and hydrocortisone [209,210,211,212]. The researchers loaded dexamethasone onto PCL bioscaffolds to achieve a sustained release of dexamethasone over a 35-day period at a concentration range of 17–163 nM which could promote macrophage phenotypic shift from M1 to M2 and promote the osteogenic differentiation of MSCs [213] (Figure 7c).

In addition to traditional anti-inflammatory drugs, emerging biologics have also shown excellent anti-inflammatory effects. For example, researchers have grafted *resveratrol* (*Res*) onto polyacrylic acid to obtain a macromolecule drug, which was then added to an acellular collagen hydrogel to create an anti-inflammatory scaffold with improved mechanical strength. In addition, the scaffold was observed to promote the proliferation and maintenance the normal phenotypic characteristics of chondrocytes and BMSCs, as well as protect them from ROS [214]. Curcumin is another common anti-inflammatory agent. Curcumin is believed to inhibit the expression of NF-κB, IL-6, and IL-11, thereby promoting an inflammatory microenvironment and inducing apoptosis in bone cancer cells to some extent [215]. Previously, curcumin was encapsulated in liposomes, and then incorporated into a 3D-printed calcium phosphate bioscaffold with design porosity. It was found that curcumin-loaded liposomes released from the 3D-printed scaffold exhibited notable cytotoxic effects on osteosarcoma cells in vitro, while promoting the viability and proliferation of osteoblasts (healthy bone cells) (Figure 7d) [216]. Furthermore, Kim et al. prepared bioscaffolds containing different concentrations of curcumin to maintain the chondrocyte phenotype and promote cartilage matrix formation [217]. 

In addition to the various anti-inflammatory drugs mentioned above, many chondroprotective drugs have also been shown to have some anti-inflammatory effects, such as chondroitin sulfate, glucosamine sulfate, hyaluronic acid, and diacerein [218,219,220,221]. Among them, the most widely studied is CS, which can regulate related enzyme activities and reduce inflammation to promote osteochondral repair [222]. Some researchers have found that fibroin–chondroitin sulfate composite bioscaffolds can reduce the IL-1β-induced inflammatory response in chondrocytes and better maintain the chondrocyte phenotype [223].

### 4.5. Other Drug-Loaded Bioscaffolds

There are numerous other drugs, although not specifically intended for osteochondral regeneration, that can promote osteochondral repair to some extent, such as insulin and platelet-rich plasma (PRP) [224]. PRP, which is abundant in numerous growth factors, proteins, and cytokines, is believed to modulate cartilage healing by stimulating cell proliferation and inducing chondrogenesis at the site of cartilage defects [225]. Bioscaffold-loaded PRP induces the polarization of M2 macrophages and facilitates the proliferation, migration, and osteogenic and chondrogenic differentiation of BMSCs [226,227]. However, thrombin can activate PRP in vivo, leading to the uncontrolled and explosive release of various growth factors. Therefore, researchers have developed a photosensitive hydrogel that enables the controlled release of growth factors through external light field manipulation. This innovative approach facilitates the proliferation and migration of chondrocytes and MSCs [228]. In addition, insulin has been found to play a surprising role in promoting the chondrocyte differentiation and osteogenic differentiation of MSCs [229,230]. By preparing a 3D-printed PCL bioscaffold modified by insulin-releasing PLGA nanodrugs, the controlled release of insulin can be achieved to promote chondrocyte proliferation and the repair of cartilage and subchondral bone [231]. 

In recent years, gene therapy has received much attention as an emerging therapy for the treatment of osteochondral injuries [232,233]. Targeted delivery gene therapy for osteochondral defects involves the precise delivery of the gene drugs necessary for osteochondral tissue repair to the damaged area through specific carriers. This approach promotes the proliferation, differentiation, and matrix synthesis of osteoblasts and chondrocytes, thereby facilitating the repair and regeneration of osteochondral defects [234,235]. Gene drug-loaded bioscaffolds are mainly categorized into bioscaffolds for loading the in vivo genes of non-viral vectors, bioscaffolds for loading the in vivo genes of viral vectors, bioscaffolds for loading the in vitro genes of non-viral vectors, and bioscaffolds for loading the in vitro genes of viral vectors [236].

**Figure 8:**
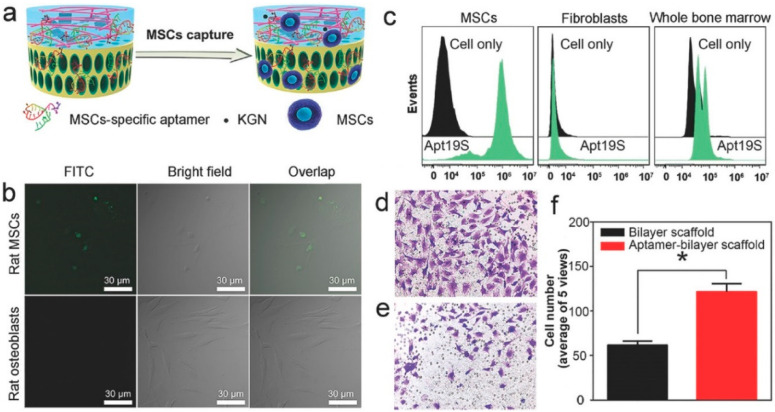
A bi-functional regeneration bioscaffold for knee repair utilizing aptamer-guided cell recruitment. (**a**) The schematic representation of MSC recruitment facilitated by the bioscaffold. (**b**) The researcher observed confocal microscopy images showing the binding of FITC-labeled Apt19S with rat MSCs and rat osteoblasts. (**c**) The researchers performed flow cytometry analysis on rat MSCs and fibroblasts. They captured a light microscopy image of the transwell assay depicting the migration of MSCs towards an (**d**) aptamer-bilayer scaffold and (**e**) bilayer scaffold. (**f**) Statistical data of the transwell assay. (* *p* < 0.05) (Reprinted with permission from Ref. [241]. Copyright 2017 John Wiley & Sons Books) FITC: fluorescein isothiocyanate; MSCs: mesenchymal stem cells.

Not only can genes be loaded onto bioscaffolds, but nucleic acid aptamers can also be utilized. Unlike genes, which are the basic units of DNA molecules, typically consisting of long DNA sequences containing information-encoding proteins, nucleic acid aptamers are short DNA or RNA molecules, often comprising tens to hundreds of base pairs, with specific sequences and structures [237]. They interact with target molecules through stable complementary pairing or specific binding, thereby affecting the expression or function of target molecules, for example, by silencing genes or modulating the activity of signaling pathways [238,239,240]. To investigate the potential of aptamers for osteochondral regeneration, researchers constructed stem cell-specific aptamer-containing bioscaffolds to recruit MSCs and promote their differentiation [241,242]. Fluorescence microscopy and flow cytometry were employed to confirm the high binding affinity of aptamers (Apt19S) to rat MSCs. The Apt19S-loaded bioscaffold attracted endogenous MSCs from the bone marrow to the injured site, and significantly repaired the osteochondral defects (Figure 8).

## 5. Drawbacks and Challenges

Drug-loaded bioscaffolds have demonstrated potential in the treatment of osteochondral defects, yet they present significant drawbacks and challenges. We identify the following four aspects of the biological applications of drug-loaded bioscaffolds as particularly difficult and challenging.

### 5.1. Controlled Drug Release in Drug-Loaded Bioscaffolds

Achieving the controlled release of drugs from drug-loaded bioscaffolds remains a challenging problem. One potential approach involves altering the scaffold’s morphology to affect drug release kinetics. Researchers have investigated the influence of the specific surface area of PLGA (poly (lactic-co-glycolic acid)) bioscaffolds with varying structures on their degradation characteristics and drug release profiles. Using minocycline as a model drug, they prepared PLGA bioscaffolds with different concentrations and morphologies. Their findings revealed that spherical bioscaffolds exhibited the highest drug-loading capacity and the most significant degree of degradation. In terms of drug release kinetics, spherical bioscaffolds showed a lower release rate at 24 h compared to the thicker and thinner wire-shaped scaffolds, likely due to their lower surface area-to-volume ratio (SVR) requiring longer diffusion times for drug release. Additionally, spherical bioscaffolds demonstrated a peak release between 14 and 21 days, potentially associated with matrix erosion [243]. Furthermore, modifying the scaffold shape, such as transitioning from hexagonal to cylindrical, has been shown to slow the release of proteins and exosomes, achieving more controlled release profiles [244]. Besides morphology, several other factors can influence the drug release kinetics of bioscaffolds. These include the scaffold’s components (metals, polymers, and composites), which affect properties like mechanical strength, hydrophilicity, and degradability, determining whether the drug is released through diffusion or degradation [245]. The composition and thickness of the scaffold’s coating also plays crucial roles. Common coatings include polymers (e.g., collagen, polydopamine), bioceramics (e.g., hydroxyapatite), and metals (e.g., gold, silver) [246,247,248,249]. For instance, Wang et al. utilized porous hydroxyapatite bioscaffolds with collagen-coated recombinant human bone morphogenetic protein-2 (rhBMP-2) delivery microspheres to achieve the controlled release of rhBMP-2 [250]. The results indicated that the biphasic release of rhBMP-2 lasted over 21 days, maintaining its osteo-inductive properties to induce osteogenic differentiation of human mesenchymal stem cells (hMSCs) in vitro. The in vivo experiments further demonstrated the scaffold’s effective bone regeneration capabilities. Additionally, layer-by-layer (LbL) polyelectrolyte multilayer films have been employed for controlled BMP-2 release. Compared to commercial collagen matrices, LbL films release less than 1% of BMP-2 within the first 3 h, which may be too rapid for preosteoblast differentiation into osteoblasts [251]. These findings underscore the importance of the release rate in promoting osteogenic differentiation. In conclusion, controlling drug release from bioscaffolds involves considering multiple factors, including scaffold morphology, material composition, and coating characteristics. Each of these elements significantly impacts the release kinetics and the scaffold’s effectiveness in therapeutic applications. 

In addition to the characteristics of the scaffold itself, the properties of the drug significantly affect drug release kinetics. Key factors include quantity, molecular weight, and solubility. A higher drug load on the bioscaffolds typically results in a prolonged release duration under the same conditions [252]. Drugs with high solubility are usually released more quickly, whereas drugs with low solubility may take longer to release [253]. The in vivo environment is a critical influence on drug release kinetics. For instance, in osteoarthritis models, bone and cartilage exist in a distinctly inflammatory microenvironment. Hyperactive inflammation can recruit immune cells, leading to increased degradation rates and significantly faster drug release [254]. To mitigate this, antioxidant and anti-inflammatory modifications can be incorporated into bioscaffolds to slow degradation and extend drug release [255]. The interaction between drugs and bioscaffolds, whether through chemical binding or physical adsorption, also impacts release kinetics [256]. Understanding these factors is essential for optimizing drug delivery systems using bioscaffolds, ensuring controlled and sustained drug release tailored to specific therapeutic needs.

Recent advancements have introduced new tools, including acoustic, optical, electrical, and magnetic modalities, to modulate the controlled release of drugs [257,258]. Kuang et al. designed a light-responsive hydrogel to promote bone regeneration in osteoporosis, achieving the controlled release of the parathyroid hormone through near-infrared light responsiveness [259]. Additionally, researchers have explored the effects of heterogeneous surface potentials on osteogenesis by doping BaTiO_3_ nanofibers into poly (vinylidene fluoride) trifluorovinyl plasmas, creating a heterogeneous distribution of surface potentials [260]. This approach enhanced mechano-transduction and promoted osteogenic differentiation of bone marrow-derived mesenchymal stem cells (BMSCs) in vitro and bone regeneration in vivo. Magnetic fields have also been utilized for remotely controlled drug release. For instance, researchers developed a magnetic sponge capsule, a magnetic porous structure that deforms rapidly and reversibly in the presence of a magnetic field, providing a controlled pumping force to release the drug from the capsule [261]. Beyond physical modalities, pH-responsive and enzyme-responsive bioscaffolds offer additional mechanisms for controlled drug release [262,263]. In summary, various intelligent strategies exist to regulate drug release from bioscaffolds for osteochondral defects. However, monitoring the drug release controlled by these methods remains challenging. Future research should aim to integrate sensors with these controlled release systems to optimize therapeutic effects.

### 5.2. Improving Biocompatibility and Safety of Drug-Loaded Bioscaffolds

**Figure 9:**
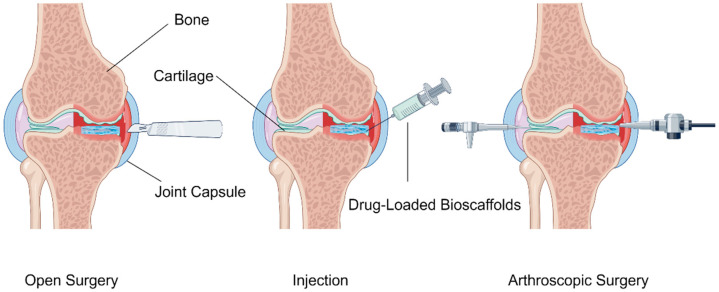
Common implantation of drug-loaded bioscaffolds for osteochondral defects. The main methods of implantation of drug-loaded bioscaffolds for osteochondral injuries include open surgery, arthroscopic surgery, and material injection. By Figdraw (https://www.figdraw.com, accessed on 24 July 2024).

Drug-loaded bioscaffolds are commonly surgically implanted to treat osteochondral defects. The main implantation methods include open surgery, arthroscopic surgery, and material injection (Figure 9) [264,265,266]. Open surgery is the most common method of implantation. This traditional method involves direct exposure of the damaged area for precise scaffold implantation. Its advantage lies in providing a clear view and ease of operation. However, it is invasive and carries a higher risk of infection [267]. The arthroscopic surgery involves inserting an arthroscope and surgical tools through a small incision to visualize and repair intra-articular injuries. It offers the benefits of minimal invasiveness, shorter recovery time, and precise visualization [268]. Superior to the above implantation methods, the material injection method involves injecting a liquid drug-loaded scaffold directly into the injury site, where it solidifies in the body to provide structural support and controlled drug release [269]. Regardless of the implantation method, local inflammation and immune responses are common. Reducing these immune responses to improve biocompatibility and long-term stability are a crucial area of investigation. 

Common tests for evaluating the biocompatibility and long-term stability of scaffolds include chemical characterization, mechanical testing, sterilization and shelf-life testing, degradation studies, drug release kinetics, cytotoxicity, biocompatibility, and histological analysis [270,271]. Given that the chemical composition of the scaffold directly influences the types of substances it releases, chemical characterization is a crucial method for assessing the safety of biological scaffolds. This involves analyzing the scaffold’s chemical composition and potential extracts or degradation products using techniques such as mass spectrometry and chromatography [272]. In addition to chemical characterization, the mechanical properties of the scaffold are significant for its long-term stability. Mechanical performance evaluations include assessing the scaffold’s tensile strength, compressive strength, and elasticity to ensure structural stability over time [273]. The chemical and mechanical properties of biological scaffolds can impact their degradation, thereby affecting their long-term stability. Researchers, therefore, monitor the scaffold’s degradation rate and by-products under physiological conditions to determine its longevity and safety [274]. For drug-loaded scaffolds, the drug is released as the scaffold degrades, making the monitoring of drug release kinetics particularly important. Drug release kinetics typically involve monitoring the release profile of the loaded drug over time to ensure consistent and controlled delivery [275]. Furthermore, before conducting in vitro studies and in vivo implantation, sterilization and shelf-life testing are essential. These tests assess the scaffold’s stability and sterility over its shelf life to extend its storage duration. Prior to in vivo implantation, the scaffold’s cytotoxicity must first be tested in vitro. In vitro cytotoxicity testing evaluates the scaffold’s effects on cell viability, proliferation, and differentiation using cell cultures to ensure its biosafety [276]. Based on favorable in vitro study results, assessing in vivo biocompatibility and long-term stability is a critical step in determining whether a biological scaffold has clinical application potential [277]. In vivo biocompatibility studies involve implanting the scaffold in animal models to observe adverse reactions, immune responses, and overall integration with host tissue [278]. Additionally, histological analysis can further evaluate tissue samples around the implanted scaffold to investigate cellular infiltration, tissue regeneration, and inflammatory responses [279].

Recent studies aim to enhance biocompatibility and safety using various strategies. For instance, natural polymers like collagen, gelatin, and chitosan, which are biocompatible, are used as matrices for drug-loaded scaffolds to treat osteochondral defects [280]. Controlling the degradation rate of bioscaffolds is another strategy to improve biocompatibility. Researchers have developed chitosan/poly(L-lactide)/pectin polyelectrolyte complex porous scaffolds that degrade slowly, avoid infection, and maintain active chondrocytes for up to 8 weeks, indicating good biocompatibility [281]. Additionally, functionalizing scaffold surfaces with exosomes to impart anti-inflammatory properties has shown promise. This approach increases resistance to inflammatory microenvironments and accelerates bone regeneration [282].

In summary, while drug-loaded bioscaffolds offer promising treatment options for osteochondral defects, ongoing research is essential to address challenges related to biocompatibility and safety. Integrating biocompatibility improvements with advanced implantation techniques can optimize therapeutic outcomes.

### 5.3. Improving Specificity for Individualized Treatment of Osteochondral Defects

Patients with osteochondral defects vary widely, with differences in the defect site, extent, and individual patient characteristics influencing treatment outcomes. Standardized drug-loaded bioscaffolds and similar technologies often fail to meet the needs of individualized treatment and must be tailored to each specific situation. Several tools allow for a degree of treatment individualization. Using 3D scanning technology, precise anatomical data can be obtained based on the shape and size of the patient’s specific defect site. This allows for the custom design of scaffolds to fit the unique anatomical features of each patient [283]. Bioscaffolds can be loaded with patient-specific cells and growth factors, such as endogenous stem cells, to avoid immune rejection and better promote osteochondral regeneration [284].

Despite these advancements, current personalized strategies face challenges such as technical complexity, high economic costs, and significant clinical operational difficulties. Future research should focus on the following aspects. Firstly, enhancing precision and efficiency, and improving the design and manufacturing techniques for personalized scaffolds. Secondly, reducing costs, developing cost-effective methods while ensuring high standards and regulation, accumulating clinical data, and gathering more clinical data to ensure the safety and efficacy of customized treatments. 

In conclusion, while individualized treatment for osteochondral defects shows promise, addressing these challenges is crucial for advancing the field and providing better patient outcomes.

### 5.4. Regulatory Challenges of Drug-Loaded Scaffolds

Given the significant clinical application prospects of drug-loaded bioscaffolds, a substantial number of these scaffolds are produced and introduced to the market annually. To ensure their safety, efficacy, and long-term stability, stringent regulatory guidelines must be established [285]. However, once bioactive scaffolds are loaded with drugs, the system becomes more complex, presenting significant challenges for regulation. Firstly, ensuring safety and biocompatibility requires extensive preclinical testing to assess toxicity, immunogenicity, and potential side effects, ensuring that the scaffold materials and loaded drugs do not cause adverse reactions in the body. Secondly, demonstrating efficacy necessitates rigorous clinical trials to prove that the scaffolds can effectively deliver drugs and achieve the desired therapeutic outcomes. Additionally, it is essential to ensure that the manufacturing process is reproducible and controlled to maintain the consistency, purity, and quality of the scaffolds. Robust quality control measures are therefore critical to ensure that each batch of drug-loaded scaffolds meets the specified standards, including continuous monitoring and testing throughout the production process. Post-market surveillance is also crucial, involving ongoing monitoring of the product after its release to identify any long-term or rare adverse effects and ensure continuous reporting and action on any safety concerns. Ethical and legal considerations are paramount during the use of scaffolds, especially when used in vulnerable populations, necessitating adherence to ethical standards in clinical trials and patient consent. Furthermore, interdisciplinary coordination among researchers, manufacturers, regulatory bodies, and healthcare providers is crucial to ensure compliance with regulatory requirements throughout the product’s lifecycle [286]. However, the regulatory framework does not always keep pace with scientific advancements. Additionally, the lack of global regulatory coordination for biomedical devices is a significant drawback [287]. Therefore, future efforts should emphasize making the commercialization of these products more accessible. 

## 6. Summary and Outlook

In conclusion, the development of drug-loaded bioscaffolds for the remediation of osteochondral defects represent a burgeoning domain within biomedical engineering and tissue regeneration disciplines. These bioscaffolds provide a platform with numerous functionalities for the site-specific administration of therapeutic agents, heralding a revolutionary advancement in osteochondral repair and regeneration strategies. Notably, the incorporation of pharmacological agents into scaffold matrices facilitates localized, prolonged release, thereby attenuating potential systemic adverse effects and furnishing a precision therapy modality. Moreover, empirical evidence underscores the efficacy of drug-loaded bioscaffolds, illustrating substantial enhancements in osteochondral tissue restoration via the modulated release of anabolic factors, anti-inflammatory compounds, and other biomolecules. Such progress intimates a transition towards more tailored and efficacious treatments for osteochondral defects, diverging from conventional approaches that frequently culminate in suboptimal healing and persistent sequelae.

Notwithstanding, the field confronts several hurdles that must be surmounted to effectuate the clinical translation of these promising technologies. These challenges encompass refining the biocompatibility and mechanical attributes of scaffold constructs, guaranteeing the enduring stability and therapeutic potency of embedded drugs, and elucidating the intricate interplays between drug-loaded bioscaffolds and the host biological milieu. Furthermore, navigating the regulatory landscape for these innovative therapeutic devices necessitates meticulous consideration to affirm their safety and therapeutic efficacy in human applications. Future investigative endeavors should concentrate on the innovation of novel scaffold matrices and drug dispensation systems, aiming for the meticulous modulation of drug release kinetics. Cutting-edge manufacturing techniques, such as additive manufacturing (3D printing), proffer exciting prospects for fabricating patient-specific bioscaffolds that precisely conform to individual anatomical and defect nuances. 

Additionally, the employment of responsive materials that can adapt to endogenous physiological cues may facilitate the dynamic modulation of drug liberation in concert with the reparative process, thereby amplifying therapeutic outcomes. In essence, cross-disciplinary collaboration among material scientists, biologists, pharmacologists, and medical practitioners is imperative for the advancement of drug-loaded bioscaffolds for osteochondral lesion therapy. By amalgamating expertise from these variegated disciplines, it is feasible to navigate extant obstacles and propel these avant-garde therapeutic modalities towards clinical fruition, potentially enhancing the management of patients with osteochondral afflictions.

## Figures and Tables

**Table 2:** Keywords that rank in the top 30 in terms of frequency.

Number	Frequency	Keywords
1	146	mesenchymal stem cells
2	116	articular cartilage
3	75	repair
4	63	chondrogenic differentiation
5	58	osteochondral defects
6	51	regeneration
7	48	tissue engineering
8	46	in vitro
9	46	bone
10	42	tissue
11	40	growth factor
12	39	autologous chondrocyte implantation
13	38	growth factors
14	33	knee
15	33	stem cells
16	33	bone marrow
17	33	differentiation
18	31	osteochondral defect
19	31	scaffolds
20	31	cartilage repair
21	29	osteoarthritis
22	28	defects
23	25	platelet-rich plasma
24	23	chondrocytes
25	23	stromal cells
26	23	osteogenic differentiation
27	23	scaffold
28	23	cartilage regeneration
29	23	in vivo
30	22	mechanical property

**Table 4:** Different nanodrugs loaded on scaffolds.

Type	Composition	Application	Reference
Metal and its oxides	Fe_3_O_4_	Palate-bone regeneration	[171]
	Ag	Bone-interfacing regeneration	[172]
	Au, Pd, γ-Fe_2_O_3_	Bone regeneration	[173]
	Au	Tissue engineering	[174]
	Co	Bone regeneration	[175]
	ZnO	Guided/bone tissue regeneration, antibacterial Osteogenic differentiation	[176]
	Cu, Zn	Bone regeneration	[177]
	MgO	Osteogenesis and angiogenesis	[178]
	Si, Zr	Bone tissue engineering	[179]
Polymers	PLA	Bone tissue engineering	[180]
	PLGA	Bone regeneration	[181]
	PCL, HA	Bone tissue engineering	[182]
	PCL, Si	Guided bone regeneration	[183]
	PDA, PEG	Rescuing cartilage degradation	[184]
	PVA	Guided bone regeneration	[185]
	SF	Osteoblast differentiation	[186]
Ceramics	HA	Osteoblast differentiation bone formation	[187]
	HA, Au, Ag	Bone regeneration	[188]
	β-TCP, Collagen, SrO	Bone tissue engineering	[189]
	ZrO_2_	Bone tissue engineering	[190]
	ZrO_2_, BaSO_4_	Enhanced bone cement properties	[191]

Fe_3_O_4_: iron(II,III) oxide (also known as magnetite), Ag: silver, Au: gold, Pd: palladium, γ-Fe_2_O_3_: gamma Iron(III) oxide (also known as maghemite), Co: cobalt, ZnO: zinc oxide, Cu: copper, MgO: magnesium oxide, Si: silicon, Zr: zirconium, PLA: polylactic acid, PLGA: poly(lactic-co-glycolic acid), PCL: polycaprolactone, HA: hydroxyapatite, PDA: polydopamine, PEG: poly(ethylene glycol), PVA: polyvinyl alcohol, SF: silk fibroin, β-TCP: beta-tricalcium phosphate, BaSO_4_: barium sulfate.
